# Mesenchymal Stromal Cell-Derived Exosomes Affect mRNA Expression and Function of B-Lymphocytes

**DOI:** 10.3389/fimmu.2018.03053

**Published:** 2018-12-21

**Authors:** Drirh Khare, Reuven Or, Igor Resnick, Claudine Barkatz, Osnat Almogi-Hazan, Batia Avni

**Affiliations:** ^1^Department of Bone Marrow Transplantation & Cancer Immunotherapy, Hadassah-Hebrew University Medical Center, Jerusalem, Israel; ^2^Department of Hematology, Hadassah-Hebrew University Medical Center, Jerusalem, Israel

**Keywords:** mesenchymal stem cells, bmMSC-derived exosomes, B-lymphocytes, next generation sequencing, ingenuity pathway analysis, CXCL8, MZB1

## Abstract

**Background:** Bone marrow mesenchymal stem cells (bmMSC) may play a role in the regulation of maturation, proliferation, and functional activation of lymphocytes, though the exact mechanisms are unknown. MSC-derived exosomes induce a regulatory response in the function of B, T, and monocyte-derived dendritic cells. Here, we evaluated the specific inhibition of human lymphocytes by bmMSC-derived exosomes and the effects on B-cell function.

**Methods:** Exosomes were isolated from culture media of bmMSC obtained from several healthy donors. The effect of purified bmMSC-derived exosomes on activated peripheral blood mononuclear cells (PBMCs) and isolated B and T lymphocyte proliferation was measured by carboxyfluorescein succinimidyl ester assay. Using the Illumina sequencing platform, mRNA profiling was performed on B-lymphocytes activated in the presence or absence of exosomes. Ingenuity® pathway analysis software was applied to analyze pathway networks, and biological functions of the differentially expressed genes. Validation by RT-PCR was performed. The effect of bmMSC-derived exosomes on antibody secretion was measured by ELISA.

**Results:** Proliferation of activated PBMCs or isolated T and B cells co-cultured with MSC-derived exosomes decreased by 37, 23, and 18%, respectively, compared to controls. mRNA profiling of activated B-lymphocytes revealed 186 genes that were differentially expressed between exosome-treated and control cells. We observed down- and up-regulation of genes that are involved in cell trafficking, development, hemostasis, and immune cell function. RNA-Seq results were validated by real time PCR analysis for the expression of CXCL8 (IL8) and MZB1 genes that are known to have an important role in immune modulation. Functional alterations were confirmed by decreased IgM production levels. Consistent results were demonstrated among a wide variety of healthy human bmMSC donors.

**Conclusion:** Our data show that exosomes may play an important role in immune regulation. They inhibit proliferation of several types of immune cells. In B-lymphocytes they modulate cell function by exerting differential expression of the mRNA of relevant genes. The results of this study help elucidate the mechanisms by which exosomes induce immune regulation and may contribute to the development of newer and safer therapeutic strategies.

## Introduction

Mesenchymal stromal cells (MSCs) are non-hematopoietic cells that have the capacity to self-renew and differentiate into various cell lineages of mesenchymal origin. MSCs can be obtained from bone marrow, adipose tissues, fetal liver, and umbilical cord blood (1–3). Bone marrow MSC (bmMSC) progenitors can be readily isolated from bone marrow and expanded *ex-vivo* without any apparent modifications in phenotype or loss of function (4). BmMSC progenitors constitutively secrete regulatory molecules and cytokines that stimulate and enhance the maturation, proliferation, differentiation, migration, and functional activation of peripheral blood mononuclear cells (PBMCs) (4–7). Several studies have demonstrated that the inhibitory effect of bmMSCs is not dependent on cell-to-cell contact. This suggests that paracrine effects, possibly by means of soluble factors, may be responsible for the interactions. The immune regulatory effects of bmMSCs have raised the possibility that they can serve as possible immune modulators in various conditions including acute myocardial infarction, ischemic stroke, acute kidney failure, Crohn's disease, and acute graft vs. host disease (aGVHD) (8, 9).

Exosomes are small membrane vesicles (30–100 nm) that are formed by a wide variety of cells, by reverse budding of the multivesicular bodies in the late endocytic compartment. Fusion of exosomes with the plasma membrane results in extracellular secretion of exosomes whose membrane is oriented the same as that of the cell (10). A number of *in vitro* as well as *in vivo* studies have demonstrated that several cell types secrete exosomes, including normal cells of hematopoietic origin such as B cells, cytotoxic T lymphocytes, and dendritic cells (11, 12). Exosomes have been found to express many types of proteins, normally on the cell surface and in plasma, cytosol, and endocytic compartment membranes. Only subsets of endosomal/lysosomal proteins are contained in exosomes and the mechanism leading to protein sorting in these multi-vesicles (MVs) is not well-understood (11–14). Several mechanical/physical interactions between exosomes and recipient cells have been reported. These include adhesion of vesicles to the recipient cell surface, internalization into endocytic compartments, and fusion with the plasma membrane and internal endosomal membranes (15).

In addition to proteins, exosomes contain RNA molecules, including messenger RNA (mRNA) and microRNA (miRNA) from the cell of origin (16, 17). The RNA can be transferred between cells and thus affects the protein production of recipient cells. Accumulating evidence indicates that exosomes play an important role in cell-to-cell communication. Several studies have shown exosomal transfer of mRNA and miRNA (17–19). These findings have led to *in vivo* studies using exosomes as a therapeutic modality. The administration of MSC-HPLC purified exosomes was shown to reduce infarct size by 17% in a mouse model (20), MSC-derived exosomes improved refractory aGVHD (20), and induced an immune regulatory response on B, T, and monocyte derive dendritic cells (21–23).

Due to the evidence of an important role of MSC-derived exosomes in immune-regulation, we evaluated the specific inhibition of human T and B-lymphocytes by bmMSC-derived exosomes and the consequent changes in mRNA expression. Importantly, we used exosomes from several MSC donors. The results of this research contribute to our understanding of MSC-derived exosome immune-modulatory mechanisms, particularly B-cell lymphocytes, and may advance the therapeutic use of these exosomes in various inflammatory diseases.

## Materials and Methods

### bmMSC Isolation and Expansion

Bone marrow (BM) aspirate was obtained from the iliac crest of 12 normal human subjects who donated BM for allogeneic transplantation, upon approval of the local Helsinki Committee (0626-15-HMO). Donors median age was 28.5 (range 20–62), six males and six females (Table S1). All subjects gave written informed consent in accordance with the Declaration of Helsinki. BM was passed through a nylon cell strainer, separated by a Ficoll-Hypaque density gradient and re-suspended in DMEM supplemented with 15% fetal bovine serum, and 1% glutamine. Onto 75 cm^2^ culture flasks, 30 × 10^6^ cells per flask were plated and maintained at 37°C in a humidified atmosphere, with 5% CO_2_. The medium was replaced twice weekly. When cells reached 80–90% confluence, they were trypsinized, re-suspended in medium supplemented with exosome depleted FBS and re-plated at a concentration of 0.25 × 10^6^ cells/75 cm^2^ flasks. FACS analysis using anti HLA-DR, CD56, CD3, and CD45 antibodies (eBioscience, USA) as negative markers and CD73, CD90 antibodies (eBioscience, USA) as positive markers, was performed.

### Isolation and Purification of Exosomes From bmMSC

Exosomes were isolated from conditioning media of MSC and cultured for 24 h in exosome depleted media, at a confluence of 80–90%. The media was collected and centrifuged successively at 300 g for 10 min and at 10,000 g for 20 min to eliminate cells and debris. Supernatant was then filtered through a 0.22 μm filter and ultra-centrifuged successively at 100,000 g for 1 h and then at 100,000 g for another hour. The pellet was suspended in a volume of 200 μl of 0.9% normal saline and stored at −20°C. Each batch of exosome was isolated from a different MSC donor (MSC passage no. 1–3).

### Characterization of bmMSC-Derived Exosomes

- **Electron Microscopy**
  Exosome pellets were loaded onto electron-microscope grids (Formvar/carbon film 200 Mesh, nickel; EMS FCF200-Ni) followed by washing, blocking, and incubating with primary antibodies (Purified Mouse Anti Human CD63, BD Pharmingen) and thereafter with gold-conjugated secondary Ab (12 nm Colloidal Gold-Affini Pure Goat Anti-Mouse IgG, EM Grade; Jackson Immuno Research). After contrasting with uranyl-acetate solution and embedding into methyl cellulose, electron micrographs were taken with Jeol (Jem-1400 Plus) transmission electron microscope (Japan) equipped with an ORIUS SC600 camera.
- **Zeta Sizer**
  Particle size was determined using the well-established dynamic light scattering method, performed with a Zeta sizer Nano Series ZEN3600F (Malvern Instruments, Malvern, UK).
- **Western Blotting**
  Purified exosomes or cells were treated with lysis buffer (Cell Culture Lysis Reagent, Promega, Wisconsin, United States) and protein concentration was determined by the Bradford assay (Sigma-Aldrich St. Louis, Missouri, United States). Proteins were separated on 4–12% SDS-PAGE gel (Invitrogen) and transferred to nitrocellulose membranes (Invitrogen). Membranes were blotted overnight with anti-human CD63 (Santa Cruz, Texas, United States) and anti-human KDEL (Santa Cruz Texas, United States), followed by appropriate HRP-conjugated secondary antibodies (Santa Cruz Texas, United States). Membranes were incubated with enhanced chemiluminescence substrate (Invitrogen, California, United States) and chemiluminescent signal was detected upon exposure to autoradiographic films.
- **FACS Assessment of Exosomes Characterization**
  Exosomes were incubated with 4 μl of 4 μm size aldehyde/sulfate latex beads 4% (w/v) (Life Technologies, California), for 15 min at room temperature, and then overnight at 4°C, under mild agitation. Functional groups remaining on the beads were blocked by incubation with glycine 100 mM for 30 min at room temperature, under mild agitation. Exosome coated beads were incubated with anti-human CD63 PerCP Cy5.5-conjugated antibodies (BD Biosciences, New Jersey, United States) and anti-human CD81 APC-conjugated antibodies (Miltenyi Biotec, Bergisch Gladbach, Germany) and analyzed by flow cytometry.

### PBMC Isolation

Peripheral blood was collected from healthy donors under approval of the local Helsinki Committee. Mononuclear cells were obtained by centrifugation over a Ficoll-Hypaque density gradient (Lymphoprep^TM^, Norway). PBMCs were washed twice with PBS and suspended in 10% exosome free serum RPMI media at a concentration of 1 × 10^6^ cells/ml.

### Exosomes Co-cultured With PHA-Stimulated PBMCs

PBMC proliferation was measured by Thymidine incorporation assay. PBMCs (1 × 10^5^) were cultured with 2 μg/ml PHA (eBiosciences Inc. San Diego, CA), in the presence or absence of purified MSC-derived exosomes (isolated from 1 × 10^6^ MSCs), in 96-well culture plates (Corning Costar, Cambridge, MA) with 0.2 ml exosome depleted medium (containing RPMI with 10% exosome-depleted FBS). After 4 days of culture, cells were pulsed for 16 additional hours with 3H-thymidine at 1 μCi/well (PerkinElmer, Boston MA, USA) and harvested. 3H-thymidine incorporation was measured using Top Count NXT (PerkinElmer, UK),

### Exosome Internalization Assays

To investigate PBMC uptake of bmMSC-derived exosomes, we incubated exosomes labeled with PKH-67 green fluorescent cell linker (Sigma-Aldrich, USA, as described in the manufacturer's protocol), with PBMCs labeled with cytoplasmic PerCP-Mouse Anti Human CD 45 (BD Biosciences, San Jose, CA). Internalization of exosomes was examined by confocal microscopy (Zeiss 710, Germany). Exosome internalization was documented at 6 h post incubation. Nuclei were stained with DAPI (Sigma-Aldrich, USA).

### T Cell Isolation and Activation

To determine the immunomodulatory effect of bmMSC-derived exosomes on T lymphocytes, *in vitro* T cell isolation using the Easysep^TM^ Human T Cell Enrichment cocktail was performed according to the manufacturer's protocol (Stem Cell Technologies, Canada). For T cell activation, desired wells were covered with anti CD3 antibodies by the addition of 50 μl of 5 μg/ml anti CD3 antibodies (Tonbo Biosciences San Diego, CA. Clone UCHT1) to each well and incubated at 37°C for 4 h. Antibody solution was then removed and 100 μl 10% exosome free RPMI media was added to each well for blocking. Cells were then washed with PBS and added at a concentration of 1 × 10^5^ T cells per well. To the desired wells, 0.4 μg of anti CD28 antibodies (Tonbo Biosciences San Diego, CA. Clone CD 28.2) were added. After 4 days of culture, cells were pulsed for 16 additional hours with 3H-thymidine at 1 μCi/well (PerkinElmer, Boston MA, USA) and harvested. Top Count NXT (PerkinElmer, UK) was used to assess 3H-thymidine incorporation.

### B Cell Isolation and Activation

To determine the immunomodulatory effect of bmMSC-derived exosomes on B-lymphocytes, *in vitro* isolation using Easysep^TM^ Human B Cell Enrichment cocktail was performed according to the manufacturer's protocol (Stem Cell Technologies, Canada). B cell activation was performed by plating isolated B cells at a concentration of 1 × 10^5^ per well in the presence of irradiated (12 Gy) PBMC, at a concentration of 3 × 10^4^ cells per well. To the desired wells, 2.5 μg/ml of a TLR7 agonist—R 848 (Enzo Life Sciences, NY, USA) and 10 ng/ml of IL2 (Peprotech, USA) were added. After 4 days of culture, cells were pulsed for 16 additional hours with 3H-thymidine at 1 μCi/well (PerkinElmer, Boston MA, USA) and harvested. Top Count NXT (PerkinElmer, UK) was used to assess 3H-thymidine incorporation. In other experiments, cells were activated for 4 days and the supernatant was collected for analysis of the secreted antibodies.

### Carboxyfluorescein Succinimidyl Ester (CFSE) Proliferation Assay

The proliferation of PBMC/lymphocyte subtypes was quantified by CFSE dilution. CFSE (Invitrogen, USA) was added to different lymphocyte subtypes at a concentration of 25 μM for 10 min at 37°C. After 3 days of incubation with PHA, MSC or exosomes, proliferation was assessed by flow cytometry.

### FACS Assessment of Exosome Attachment to Different PBMCs

To investigate the uptake of bmMSC-derived exosomes by different PBMC populations, green fluorescent cell linker PKH-67 labeled exosomes (Sigma-Aldrich, USA) were incubated with PBMC. Twenty-four hours later, PBMCs were labeled with APC- Anti Human CD 19 (Becton Dickinson, Ireland), APC- Anti Human CD 3 (Biolegend, USA) and PE—Anti Human CD 14 (eBioscience, USA) and assessed by flow cytometry. The viability of PBMCs after 24 h incubation with PKH labeled exosomes was determined by the addition of 5 μg/ml propidium iodide (PI) and was found to be above 95%.

### Lymphocyte RNA Extraction and Library Preparation

Lymphocyte RNA was extracted using the trizol method. For quality control of RNA extraction yield and library synthesis products, RNA Screen Tape kit (Agilent Technologies, Waldbronn, Germany), D1000 ScreenTape kit (Agilent Technologies, Waldbronn, Germany), Qubit® RNA HS Assay kit (Invitrogen, USA), Qubit® DNA HS Assay kit (Invitrogen, USA) were used for each specific step. For mRNA library preparation, KAPA Stranded mRNA-Seq Kit with mRNA Capture Beads (Kapabiosystems, KK8421, https://www.kapabiosystems.com) was used. In brief, one μg was used for the library construction; the library was eluted in 20 μl of elution buffer. Libraries were adjusted to 10 Mm; 10 μl, 50% from each sample was collected and pooled in one tube. Multiplex samples Pool (1.5 pM including PhiX 1.5%) were loaded on NextSeq 500/550 High Output v2 kit (75 cycles) cartridge (Illumina, USA) and loaded on the NextSeq 500 System (Illumina, USA), with 75 cycles and single-Read Sequencing conditions.

### Statistical Analysis

*In vitro* results represent mean values of at least three experiments. In each experiment, duplicate or triplicate samples were performed. *P*-values were calculated using Student's *t-*test. SDs and *p*-values in all *in vivo* experiments were calculated by Student's *t*-test using Prism software. *P* < 0.05 was considered statistically significant.

### Bioinformatics

#### Trimming and Filtering of Raw Reads

The NextSeq basecall files were converted to fastq files using the bcl2fastq (v2.17.1.14) program with default parameters. Following quality-trimming, adapter sequences were removed with cutadapt (version 1.11, http://cutadapt.readthedocs.org/en/stable/), using a minimal overlap of 1 (-O parameter), allowing for read wildcards, and filtering out reads that became shorter than 15 nt (-m parameter). The remaining reads were further filtered to remove very low-quality reads, using the fastq_quality_filter program of the FASTX package (version 0.0.14, http://hannonlab.cshl.edu/fastx_toolkit/), with a quality threshold of 20 at 90 percent or more of the read's positions.

#### Mapping and Differential Expression Analysis

The processed fastq files were mapped to the human transcriptome and genome using TopHat (v2.0.14). The genome version was GRCh38, with annotations from Ensembl release 84. Uniquely mapped reads were quantified using htseq-count (version 0.6.0, http://www-huber.embl.de/users/anders/HTSeq/doc/count.html). Strand information was set to “reverse”, and an annotation file was used that lacked information for genes of type IG, TR, Mt, rRNA, tRNA, miRNA, misc_RNA, scRNA, snRNA, snoRNA, sRNA, scaRNA, piRNA, vaultRNA, ribozyme, artifact, and LRG_gene.

Normalization was done and differential expression analyzed with the DESeq2 package (version 1.12.4). Since, the effect of activated vs. non-activated cells masked the effect of exposure to exosomes, an additional analysis was performed without the non-activated samples. This latter analysis showed sample B7 (activated B-lymphocytes incubated with exosomes) to be an outlier, and it was thus removed. Genes with a sum of counts <10 over all samples were filtered out prior to normalization, then size factors and dispersion were calculated. Differential expression, between the duplicate activated B-lymphocytes and the remaining five samples of B-lymphocytes incubated with exosomes, was calculated with default parameters. The significance threshold was taken as padj <0.05.

#### Target Prediction and Pathway Analysis

QIAGEN's Ingenuity® Pathway Analysis (IPA®, QIAGEN Redwood City, www.qiagen.com/ingenuity) was applied to the genes that were differentially expressed (padj < 0.1), and compared among activated lymphocytes, between those incubated and not incubated with bmMSC-derived exosomes, to identify enriched canonical pathways, molecular functions and diseases.

#### Real-Time PCR Analysis

B cell total RNA was isolated with TRIzol reagent (Invitrogen, San Diego, CA, USA) according to the supplier's protocol. RNA quality and quantity were determined by gel electrophoresis and photometry. One microgram of total RNA was used to synthesize cDNA using the High-Capacity cDNA kit (Applied Biosystems, Waltham, MA, USA) following the supplier's instructions. Relative expressions of CXCL8 and MZB1 were detected using the TaqMan Gene Expression Assay Kit (Applied Biosystems, Waltham, MA, USA), with GAPDH as a reference gene. All primers were purchased from Applied Biosystems (Waltham, MA, USA). Real-Time PCR reactions were conducted using StepOne Plus (Applied Biosystems, Waltham, MA, USA). Data were analyzed by StepOne Software version 2.2 (Applied Biosystems, Waltham, MA, USA).

**Table:** 

**Gene symbol**	**Species**	**Assay ID**
CXCLl8	Human	Hs00174103
MZB1	Human	Hs00414907
GAPDH	Human	Hs02786624

#### Immunoglobulin Estimation by ELISA

Supernatant was collected from cultures of activated B-lymphocytes with bmMSC-derived exosomes, for immunoglobulin analysis. IgG, IgA, and IgM levels were examined using the eBiosciences ELISA kit (eBiosciences, USA) according to the manufacturer's instruction manual. In brief, required numbers of wells were coated with 100 μl of capture antibodies with a coating buffer, and plates were sealed and incubated overnight at 4°C. The next day, the liquid phase was aspirated, plates were washed and then incubated at room temperature (RT) for 2 h, after the addition of 250 μl of blocking solution. Plates were washed and 100 μl of supernatant media was pipetted from each of the pre-diluted standards, controls and cell cultures (in duplicates) and incubated at RT for 2 h. After another washing step, 100 μl of diluted detection antibody was added to each well, and plates were incubated at RT for 1 h. Another washing step was done and a 100 μl of substrate solution was added to each well. Plates were incubated for 15 min at RT followed by the addition of 100 μl of stop solution. Absorbance was measured at 450 nm wavelength and 570 nm readings. The amount of IgG, IgA, and IgM in the culture medium sample was calculated from the standard curve.

## Results

### bmMSC Inhibition of Mononuclear Cell Proliferation

**Figure 1:**
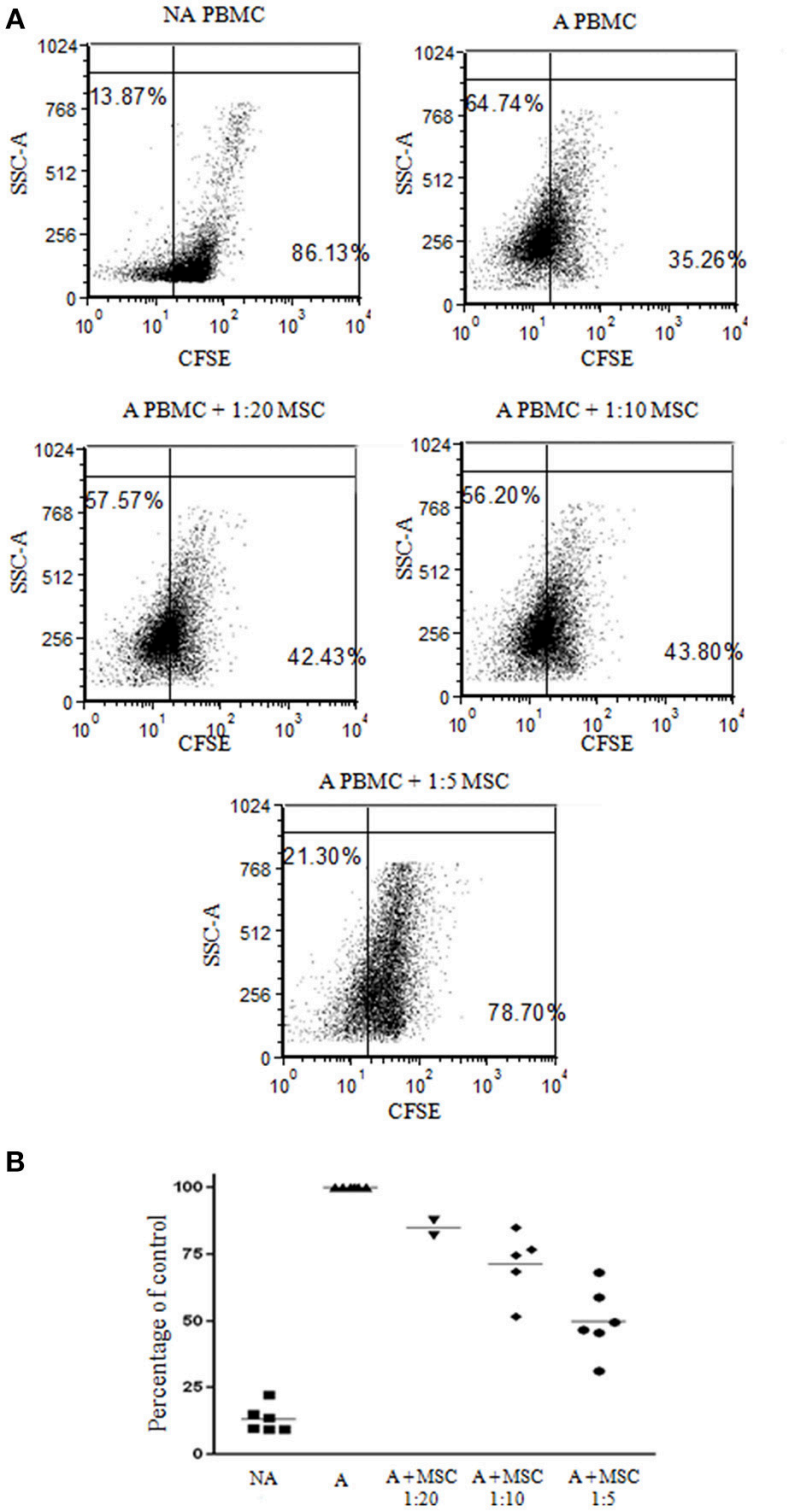
Inhibition of peripheral blood mononuclear cell (PBMC) proliferation by mesenchymal stem cells (MSCs). Carboxyfluorescein succinimidyl ester (CFSE) labeled 1 × 10^5^ PBMCs were activated with 2 μg/ml PHA and incubated for 4 days with and without MSCs at a ratio of 1:20, 1:10, and 1:5. **(A)** A representative FACS analysis of CFSE-labeled PBMCs, activated and non-activated, with and without MSCs at different ratios. **(B)** A graphic presentation of the percent suppression of activated PBMCs with different MSCs at a ratio of 1:20, 1:10 (*p* < 0.003), and 1:5 (*p* < 0.001). Data are expressed as the mean of tested samples. Activated condition counts were set to represent 100%.

Bone marrow was obtained from 12 healthy donors (Table S1) and cultured for bmMSC cells. Cells from each culture were characterized by FACS analysis using anti HLA-DR, CD56, CD3, and CD45 antibodies as negative markers and CD73 and CD90 antibodies as positive markers (Figure S1). The inhibitory effect of the bmMSC cells on immune cell proliferation upon activation was confirmed by *in vitro* CFSE proliferation assays. In our experiments the proliferation of PHA-stimulated PBMCs (Figure 1) was significantly inhibited when cultured with bmMSC; proliferation was reduced by 50, 28, and 15%, depending on the PBMC/MSC ratio (1:5, 1:10, and 1:20, respectively). Similar results were obtained in thymidine uptake experiments (Figure S2).

### bmMSC and Exosome Characterization

**Figure 2:**
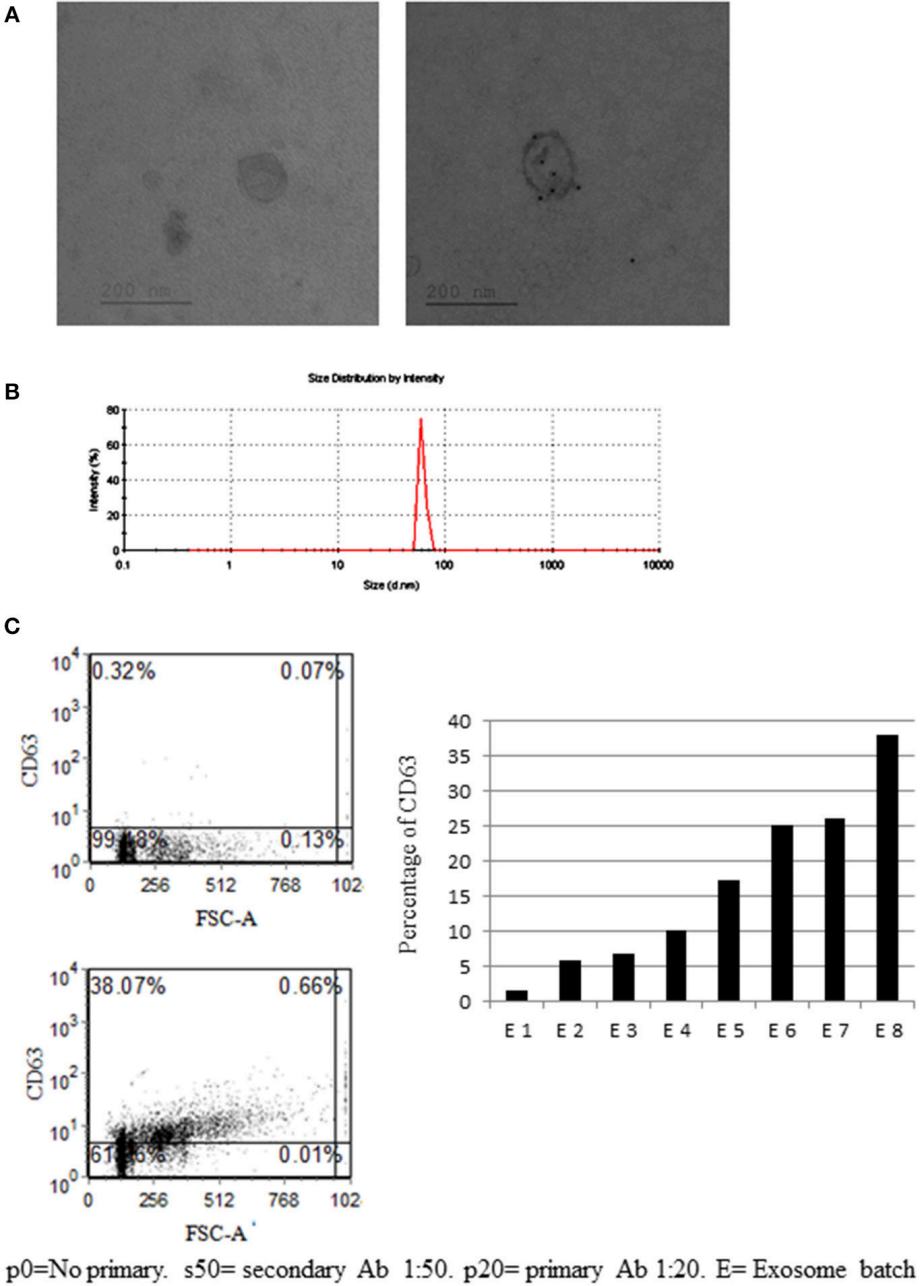
Mesenchymal stem cell (MSC)-derived exosome characterization. **(A)** Electronic microscopy of MSC-derived exosomes. Right image—no primary antibody (Ab). Right image—exosome stained with gold-conjugates, which are secondary antibody to anti-CD63. **(B)** Zeta sizer measurement of MSC-derived exosomes. (A representative measurement of 4 extractions is shown). **(C)** Anti-CD63 labeled exosomes bound to aldehyde sulfate beads. Right image—a representative graph of the percentage of CD63 staining in eight exosome batches, isolated from 8 MSC donors. The difference in CD63 expression might reflect the variability between donors and protein expression in their MSCs.

From bmMSC culture medium, bmMSC-derived exosomes were isolated, using serial centrifugations. Each batch of exosome was isolated from a different MSC donor. The presence of isolated bmMSC-derived exosomes was established by electron microscopy imaging (Figure 2A). The average size of the MSC-derived exosomes was evaluated by Zeta Sizer. The size range of the MSC-derived micro-particles was 65–100 nano-meters, as expected for secreted exosomes (Figure 2B). The expression of CD63 on exosomes from 8 MSC donors was validated by FACS analysis following binding to aldehyde sulfate beads (Figure 2C). CD81 expression was shown by FACS analysis on exosomes from 5 MSC donors (Figure S3B). The absence of KDEL (an ER protein) was shown using western blot analysis (Figure S3A).

### bmMSC-Derived Exosomes Inhibit the Proliferation of PBMCs and Lymphocyte Subsets

**Figure 3:**
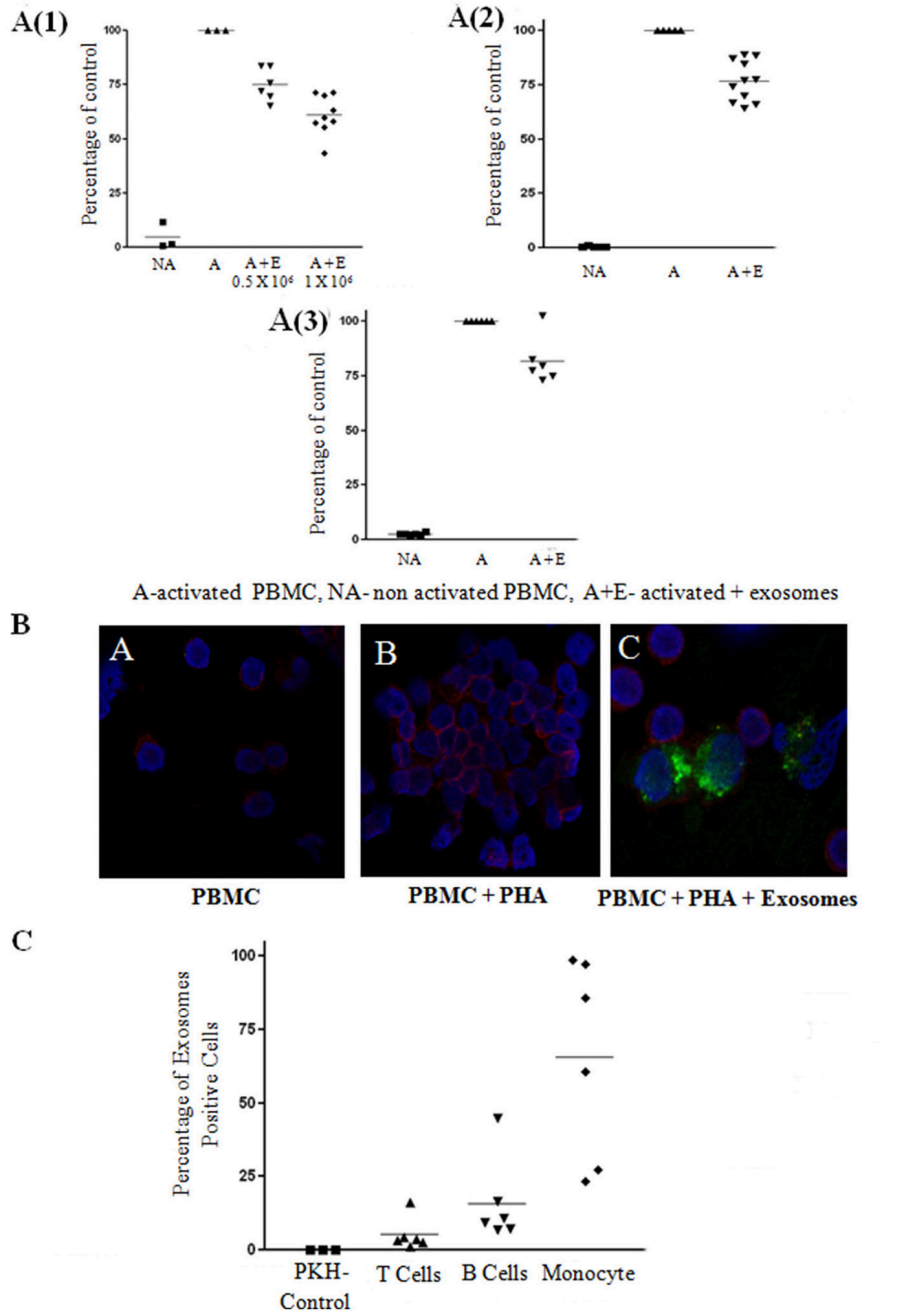
Exosome suppression of activated peripheral blood mononuclear cell (PBMCS) and lymphocytes and internalization assays. **(A)** Thymidine incorporation assays. Mesenchymal stem cell (MSC)-derived exosomes co-cultured with: **(1)** PHA-activated PBMCs. The results summarize 3 experiments with 6 batches of exosomes derived from 0.5 × 10^6^ MSCs and 9 batches of exosomes derived from 1 × 10^6^ MSCs (batches no. 1–9). Control—Activated condition counts set to represent 100%. *P* < 0.001. **(2)** CD3/CD28 activated T-lymphocytes. The results summarize 5 experiments with 11 batches of exosomes derived from 1 × 10^6^ MSCs (batches no. 1–11). Control—Activated condition counts were set to represent 100%, *p* < 0.001. **(3)**. R-848/IL2 activated B-lymphocytes. The results summarize 3 experiments with 6 batches of exosomes derived from 1 × 10^6^ MSCs (batches no. 4–9). Control—Activated condition counts were set to represent 100%, *p* < 0.004. **(B)** The internalization of exosomes by activated lymphocytes was assessed by confocal microscopy: Blue color, nucleus; Red color, cytoplasmic CD45; Green color, exosomes. **(C)** FACS analysis of PKH positive peripheral blood mononuclear cell, gated to different cell populations. The results summarize 3 experiments with 6 batches of exosomes. Percent uptake of 0.04%, 5.22%, 15.89%, 65.49% in PKH only, T cell, B cell, monocyte group, respectively.

To investigate the immune regulatory function of bmMSC-derived exosomes, we examined the effect of these exosomes on the proliferation of activated PBMCs and various lymphocyte subsets. First, the proliferation of PHA stimulated human PBMCs in the presence of bmMSC-derived exosomes at different concentrations (exosomes isolated from 1 × 10^4^ MSC cells, 1 × 10^5^ cells, 1 × 10^6^ cells) was analyzed by the thymidine incorporation assay. We found significant inhibition of proliferation in the presence of MSC-derived exosomes isolated from 1 × 10^5^ and 1 × 10^6^ MSC. The proliferation of PHA–stimulated PBMCs co-cultured with MSC-derived exosomes isolated from 1 × 10^6^ MSC was reduced by 36.72% (Figure 3A1). For further experiments, we used MSC-derived exosomes isolated from 1 × 10^6^ MSC.

Next, we isolated T and B-lymphocyte populations, and examined the effect of the exosomes on the activation of these cells. Proliferation of 1 × 10^5^ isolated T cells activated with anti CD3/CD28 in the presence of MSC-derived exosomes was reduced by 23.17% (Figure 3A2) and proliferation of isolated 1 × 10^5^ B cells activated with R-848/IL2 co-cultured with MSC-derived exosomes was reduced by 18.33% (Figure 3A3).

### Assessment of MSC-Derived Exosome Uptake by PBMCs

Since we found an inhibitory function of MSC-derived exosomes on PBMCs, as well as on isolated B and T lymphocytes, we evaluated the uptake of bmMSC-derived exosomes by PBMCs. For this purpose, bmMSC-derived exosomes labeled with PKH-67 dye were incubated for 6 h with PBMCs labeled with cytoplasmic CD45 dye. Figure 3B shows PKH-67 green-labeled exosomes surrounding PerCP red labeled lymphocytes.

The uptake of bmMSC-derived exosomes by different PBMC populations was assessed by FACS analysis of PKH positive cells gated to CD3, CD19, and CD14 positive cells. As seen in Figure 3C, bmMSC-derived exosomes were mainly found in monocytes and B cell lymphocytes. A flow cytometry gating strategy to identify the three subsets (B cells, T cells, and monocytes) of PBMCs is shown in Figure S4.

### Assessment of the Lymphocyte mRNA Expression Profile After Incubation With MSC Derived Exosomes

**Figure 4:**
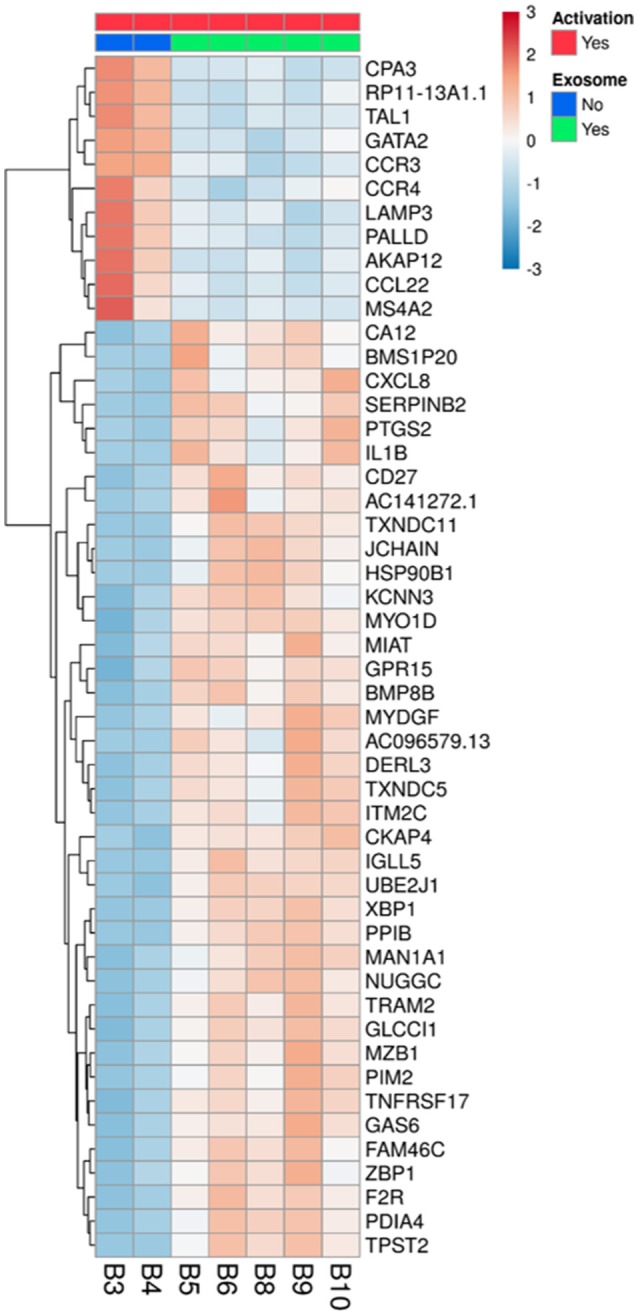
Differentially expressed genes in activated B-lymphocytes, with or without incubation with exosomes. All 5 replicates of cells exposed to exosomes (batches no. 6, 7, 8), samples B5, B6, B8, B9, and B10 (green), were compared to the duplicate samples of cells without exosome exposure, B3 and B4 (blue). The normalized expression of significantly expressed genes, padj <0.05, is shown as a heatmap, after scaling the values for each gene. The color scale is indicated on the top right corner (blue, below average; red, above average). Genes are ordered by hierarchical clustering. Only the 50 genes that were most differentially expressed are shown, with their official symbols.

To understand the biological changes that occur in lymphocytes following treatment, we extracted mRNA from non-activated B-lymphocytes, activated B-lymphocytes and activated B-lymphocytes incubated with bmMSC-derived exosomes from 3 donors, in duplicates. Using the Illumina sequencing platform, mRNA profiling was performed and differential expression was calculated using DESeq2. Comparing all groups, activation showed an extensive effect on mRNA expression, which involved thousands of genes, thus masking the subtler effect of exposure to exosomes. Inspecting the expression patterns without the non-activated B-lymphocytes enabled the identification of 186 genes with significantly different expression in the activated lymphocytes, between those incubated and not incubated with exosomes (combined as one group) (Table S2 and Figure 4). Applying the IPA® software to this list of genes, the two most significantly altered bio-functions were: cell-to-cell signaling and interaction and cellular movement (Tables S3, S4). Canonical pathway analysis has shown effects on the TH2 pathway, IL8 signaling, TREM1 signaling, colorectal cancer metastasis signaling, NFAT in regulation of the immune response, and IL-17F in allergic inflammatory airway diseases (Table 1).

**Table 1:** Enrichment of IPA® canonical pathways.

**Ingenuity canonical pathways**	**–log (B-H *p*-value)**	**B-H *p*-value**	**Ratio**	**Molecules**
Granulocyte adhesion and diapedesis	5.87	1.35E-06	0.0791	CXCL9, CCL7, IL1B, CXCL10, CXCL8, HSPB1, CCL22, MMP19, HRH4, CXCL1, CSF3, CXCL3, CXCL5, MMP1
Role of IL-17A in psoriasis	4.75	1.78E-05	0.385	S100A9, CXCL1, CXCL3, CXCL5, CXCL8
Granulocyte adhesion and diapedesis	4.2	6.31E-05	0.0635	CXCL9, CCL22, CCL7, MMP19, IL1B, CXCL10, CXCL1, CXCL3, CXCL5, MMP1, ITGB7, CXCL8
Hepatic fibrosis/hepatic stellate cell activation	3.63	0.000234	0.0601	CCR7, CXCL9, IL1B, VEGFA, CD14, MET, COL4A4, CXCL3, COL18A1, MMP1, CXCL8
Role of IL-17F in allergic inflammatory airway diseases	3.58	0.000263	0.136	CCL7, IL1B, CXCL10, CXCL1, CXCL5, CXCL8
Role of IL-17A in arthritis	3.58	0.000263	0.104	CCL7, PTGS2, CXCL1, CXCL3, CXCL5, MMP1, CXCL8
Differential regulation of cytokine production in macrophages and T helper cells by IL-17A and IL-17F	2.95	0.001122	0.222	IL9, IL1B, CXCL1, CSF3
Airway pathology in chronic obstructive pulmonary disease	2.72	0.001905	0.375	CXCL3, MMP1, CXCL8
Differential regulation of cytokine production in intestinal epithelial cells by IL-17A and IL-17F	2.61	0.002455	0.174	IL9, IL1B, CXCL1, CSF3
Th1 and Th2 activation pathway	2.43	0.003715	0.0486	GATA3, DLL1, IL9, ICOS, IL24, LTA, CCR4, MAF, CCR3
Th2 pathway	2.38	0.004169	0.0533	GATA3, DLL1, IL9, ICOS, IL24, CCR4, MAF, CCR3
IL-17 signaling	2.26	0.005495	0.0706	CXCL10, PTGS2, CXCL1, IL19, CXCL5, CXCL8
Role of tissue factor in cancer	2.24	0.005754	0.0574	GNAQ, IL1B, VEGFA, CXCL1, MMP1, CXCL8, P4HB
Communication between innate and adaptive immune cells	2.21	0.006166	0.0674	CCR7, TNFRSF17, IL1B, CXCL10, TNFRSF13B, CXCL8
Oncostatin M signaling	2.16	0.006918	0.118	CHI3L1, MT2A, EPAS1, MMP1
Inhibition of matrix metalloproteases	1.95	0.01122	0.103	MMP19, ADAM12, TFPI2, MMP1
Role of hypercytokinemia/hyperchemokinemia in the pathogenesis of influenza	1.82	0.015136	0.093	IL9, IL1B, CXCL10, CXCL8
IL-17A signaling in gastric cells	1.54	0.02884	0.12	CXCL10, CXCL1, CXCL8
Bladder cancer signaling	1.54	0.02884	0.0575	MMP19, VEGFA, THBS1, MMP1, CXCL8
Role of cytokines in mediating communication between immune cells	1.54	0.02884	0.0741	IL1B, IL24, CSF3, CXCL8
Unfolded protein response	1.54	0.02884	0.0741	HSPA5, XBP1, DNAJB9, P4HB
Intrinsic prothrombin activation pathway	1.42	0.038019	0.103	THBD, F5, COL18A1
Pathogenesis of multiple sclerosis	1.39	0.040738	0.222	CXCL9, CXCL10

**Figure 5:**
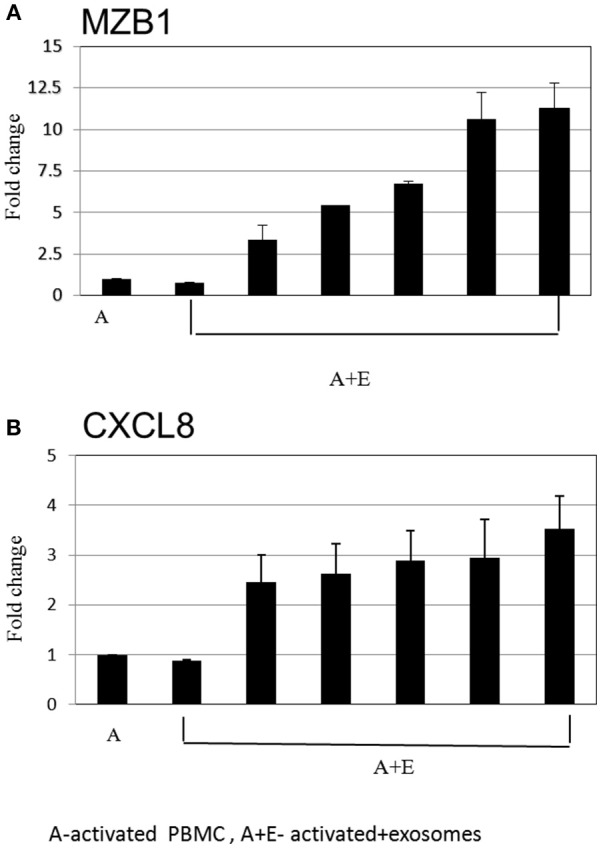
Real time PCR analysis. Expression levels of mRNA depicted for **(A)** MZB1 **(B)** CXCL8, were measured in activated B cells with or without exosomes. The graphs show the results of 6 exosome batches (MZB1 exosome batches 6, 8, 10, 12, 9, 3, respectively; CXCL8 exosome batches 6, 9, 10, 8, 12, 3, respectively). GAPDH was used as internal controls for targeting mRNA expression, and data are expressed as the mean of triplicate samples ± S.E.

After inspection of the list of the 50 genes that were most differentially expressed (11 downregulated and 39 upregulated) (Figure 4) and molecules that are involved in the canonical pathways identified by the IPA® software (Table 1), we selected two biologically relevant genes, CXCL8 and MZB1. The upregulation of these genes in B cells upon treatment with MSC-derived exosomes was verified by real time PCR analysis. Relative expression of CXCL8 and MZB1 was detected using the TaqMan Gene Expression Assay Kit, with GAPDH as a reference gene. As seen in Figure 5, in correlation with RNA-Seq results, both CXCL8 and MZB1 expression levels were found to be elevated in 5 of 6 exosome batches used.

### Immunoglobulin Estimation by ELISA

**Figure 6:**
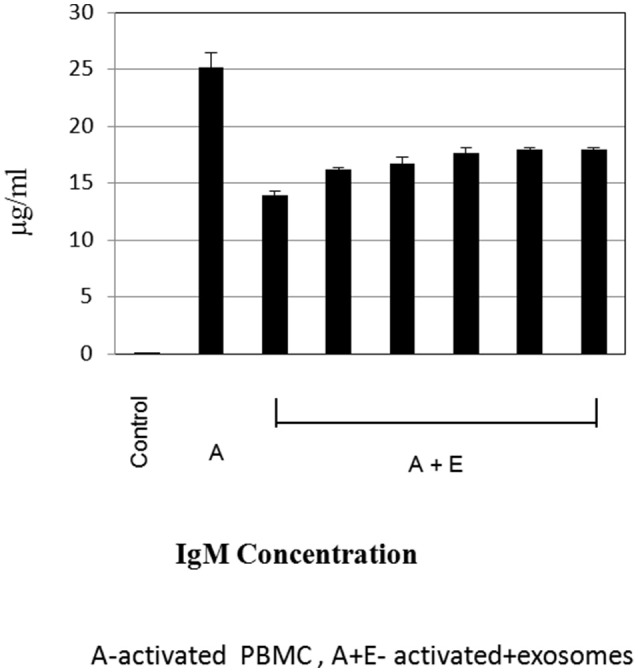
Immunoglobulin estimation by ELISA. Expression levels of immunoglobulin M were measured by ELISA, in cell culture supernatant of activated B-lymphocytes cultured with or without exosomes. The graphs show the results of 6 exosome batches (batches no. 9, 8, 3, 10, 6, 12), cultured with the same single B lymphocyte donor. Data are expressed as the mean of triplicate samples ± S.E.

To further evaluate the immune regulatory effect of MSC-derived exosomes on B- lymphocyte function, supernatant was collected from cultures of activated B- lymphocytes (1 × 10^5^ per well) with bmMSC-derived exosomes (isolated from 1 × 10^6^ MSCs), for immunoglobulin analysis. As shown in Figure 6, the addition of bmMSC-derived exosomes to activated B cells significantly inhibited the production of IgM, as tested by the ELISA assay, while no significant change was observed in the IgG and IgA levels (Figure S5). IPA analysis identified 6 genes that were significantly altered and that affect IgM levels: JCHAIN, PTGS2, POU2AF1, TNFRSF13B, SH2D1A, and LTA.

## Discussion

Increasing evidence indicates that exosomes play an important role in cell-to-cell communication; this has led to investigations of the immune regulatory effects exerted through exosome secretion (15, 24). We evaluated the specific inhibition of lymphocytes by *in vitro* bmMSC-derived exosomes and particularly their effect on B cell-specific mRNA expression.

Quantification of exosomes is a problematic issue. Like several other investigators, in all the experiments we performed, we used the number of producing cells (exosomes derived from 1 × 10^6^ MSCs) for quantification. Our data show that the levels of staining with CD63 varied in MSC-derived exosomes according to donors. This may imply a difference in the level of exosome secretion or in the level of CD63 expression of exosomes from different donors. Variation between donors is expected, in contrast to experiments that involve different cell lines or mice.

Using bmMSC-derived exosomes from several donors, at different concentrations, we showed a maximal 36.72% suppression in the proliferation of activated PBMCs. These findings correlate with the observation that MSCs suppress the immune response more than do their MVs (25). However, this could be due to the MV isolation method and the difficulty in matching the concentration of MSCs and the derived MVs used.

From several repeated experiments, using a wide variety of MSCs and their derived exosomes, we obtained suppression of 23.17% of the T-cell population and 18.33% of the B-cell population. Similar to Trapani et al.'s (26) findings, FACS assessment of exosome uptake by the various mononuclear populations showed a significant differential uptake between monocyte, B cell, and T cell populations (65.49, 15.89, and 5.22%, respectively). The effect of exosome uptake on monocyte behavior is currently being studied by our group. Indeed, no direct correlation between uptake proportion and immunosuppression level was found. This may indicate different functional effects of MSC-derived exosomes on different subsets of the immune system. We are currently studying the correlation between uptake proportion and immunosuppressive effect by examining the various effects of exosomes on other subsets of the immune system.

A number of studies indicate that MSC-derived exosomes exert their effect via horizontal transfer of proteins, mRNAs and regulatory microRNA (27). Collino et al. (18) showed selective over-representation of miRNA in the MVs associated to targets involved in the immune system processes, including leukocyte activation and differentiation. Based on this evidence of the possible transference of miRNA to target cells, we investigated activated B-cell mRNA changes after incubation with bmMSC-derived exosomes. Using the Illumina sequencing platform for mRNA profiling, activation showed an extensive effect on mRNA expression, as expected, thousands of genes were affected. Focusing on activated B-lymphocytes revealed 186 genes with significantly different expression in those incubated with exosomes.

Cell-to-cell signaling and interaction, and cellular movement pathways, were significantly altered after the addition of bmMSC-derived exosomes, as demonstrated by the IPA® software. This strengthens the already well-established understanding that immune regulation is dependent on the interaction between cellular subsets of the immune system. To our knowledge, this is the first time that mRNA differential expression profiling was performed on targeted immune cells regulated by bmMSC-derived exosomes.

From the 50 genes that were most differentially expressed, and considering the molecules that were involved in the canonical pathways identified by the IPA® software, we identified 11 downregulated genes that have important roles in cell trafficking, development, hemostasis, and immune cell function (e.g., CCR3, CCR4, and CCL22) in the exosome incubated cells. This correlates to Corcione et al.'s (5) findings that showed MSC to induce decreased chemokine receptor expression on B-lymphocytes with significantly reduced B-lymphocyte chemotaxis. Additionally, in the exosome incubated cells, we identified 39 upregulated genes that have important roles in immune regulation. SerpinB2 was found to have an important role in Th1 response (28), with increased Th1 response in SerpinB2 knockout mice. PTGS2 is a key enzyme in prostaglandin biosynthesis, PGE2 has been implicated as a powerful endogenous immunosuppressive agent that inhibits T cell proliferation and IL2 production (29). We validated our RNA-Seq results by real time PCR analysis of two interesting genes that are important in immune modulation: CXCL8 (IL8) and MZB1. The IL8 molecule was identified by the IPA® software to be involved in several canonical pathways. Moreover, it was reported to have an important role in attracting myeloid-derived suppressor cells, which are known to inhibit T cell activation and proliferation (30). MZB1 has been found to control CA^+2^ hemostasis, resulting in decreased cell proliferation and BCR-mediated CA mobilization in certain subsets of B-lymphocytes (31). These results further strengthen the hypothesis that immune suppression modulated by MSC-derived exosomes relies on interactions between various elements of the immune system.

MSCs were reported to have both inhibitory (32) and stimulatory effects (33) on B-cell proliferation differentiation, and antibody production. Similar to Budoni et al. (22), we have shown that IgM production levels were decreased, further supporting the inhibitory effect of MSC exosomes on B-lymphocytes. Due to discrepancies reported regarding the immunomodulatory effect of MSCs, it is important to obtain bmMSCs and their counterpart exosomes from different sources, to ensure consistency. This was a strength of the current study, in which bmMSCs and exosomes were obtained from a number of healthy human bmMSCs donors. Overall, functional consistency was shown in results between the donors.

In conclusion, we demonstrated that exosomes inhibit the proliferation of several types of immune cells, and B-lymphocytes in particular. The differential expression of mRNA affects cell trafficking, development, hemostasis, and immune cell function. Understanding the mechanisms by which exosomes are involved in immune regulation may help in the development of therapeutic strategies that avoid the administration of MSCs. This may circumvent some of the safety issues related to the use of living cells, such as the risk of transformation of the cells. Lack of histocompatibility antigen expression will enable repeated administration without eliciting an immune response.

## Availability of Data and Materials

All data generated or analyzed during this study are included in this published article (and its Supplementary Information Files).

## Author Contributions

BA and OA-H designed and supervised the experiments, analyzed the data, critically revised the article for important intellectual content, and wrote the manuscript. DK performed the experiments, analyzed the data, and wrote the manuscript. RO contributed in financial support and final approval of the manuscript. IR contributed technical support and data analysis. CB contributed technical support. All authors read and approved the final manuscript.

### Conflict of Interest Statement

The authors declare that the research was conducted in the absence of any commercial or financial relationships that could be construed as a potential conflict of interest.
