# Supplementary material for: Mesenchymal Stromal Cell-Derived Exosomes Affect mRNA Expression and Function of B-Lymphocytes
Source: Front Immunol. 2018 Dec 21;9:3053. doi: 10.3389/fimmu.2018.03053 (PMC6308164; doi:10.3389/fimmu.2018.03053)
Supplement: Supplementary file 1 [file Table_1.DOCX]

| **Ethnicity** | **Gender** | | **Age** | | **Donor no.** |
| --- | --- | --- | --- | --- | --- |
| Arab | | Female | | 23 | **1** |
| Arab | | Female | | 22 | **2** |
| Jew | | Male | | 20 | **3** |
| Arab | | Male | | 41 | **4** |
| Jew | | Female | | 20 | **5** |
| Arab | | Female | | 29 | **6** |
| Arab | | Male | | 22 | **7** |
| Jew | | Male | | 44 | **8** |
| Un known | | Male | | 62 | **9** |
| Jew | | Female | | 28 | **10** |
| Jew | | Male | | 37 | **11** |
| Arab | | Female | | 29 | **12** |

**Table S1:** Donors characteristics
