# Supplementary figures and images for: Mesenchymal Stromal Cell-Derived Exosomes Affect mRNA Expression and Function of B-Lymphocytes
Source: Front Immunol. 2018 Dec 21;9:3053. doi: 10.3389/fimmu.2018.03053 (PMC6308164; doi:10.3389/fimmu.2018.03053)

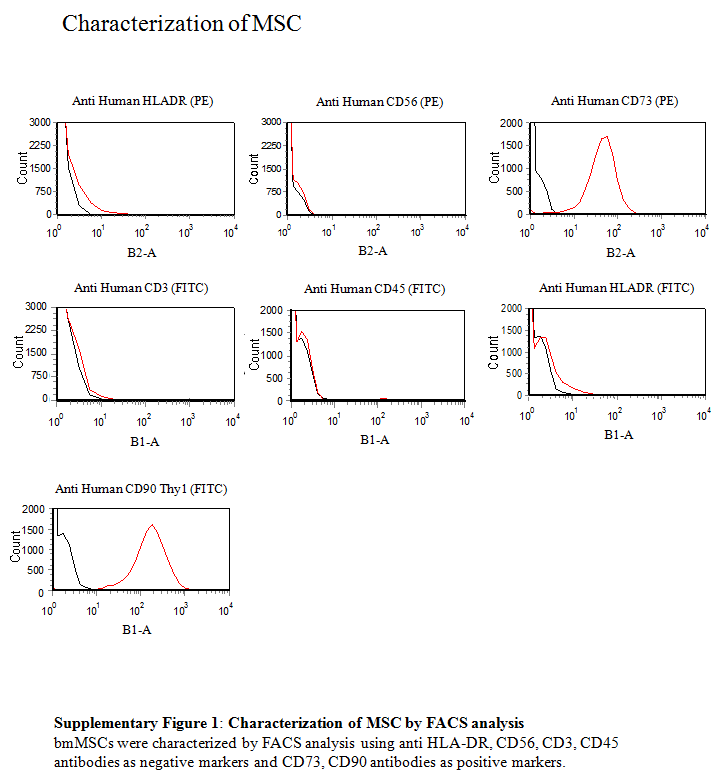

Supplement: Supplementary file 5 [file Image_1.TIF]

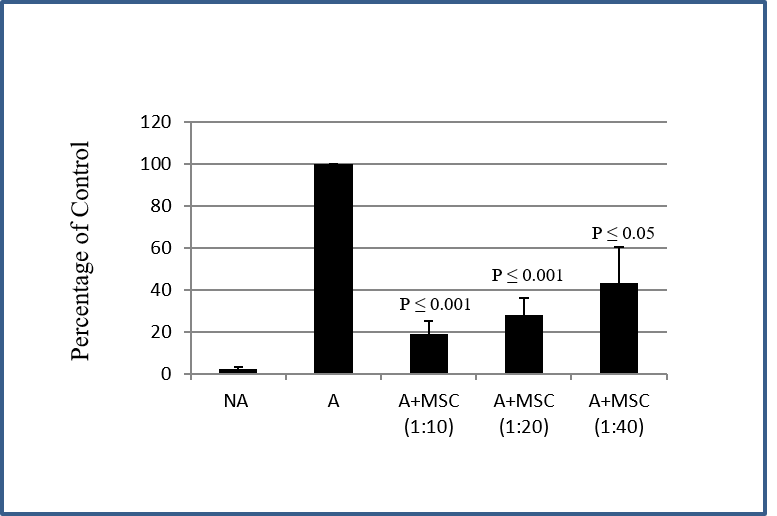

Supplement: Supplementary file 6 [file Image_2.TIF]

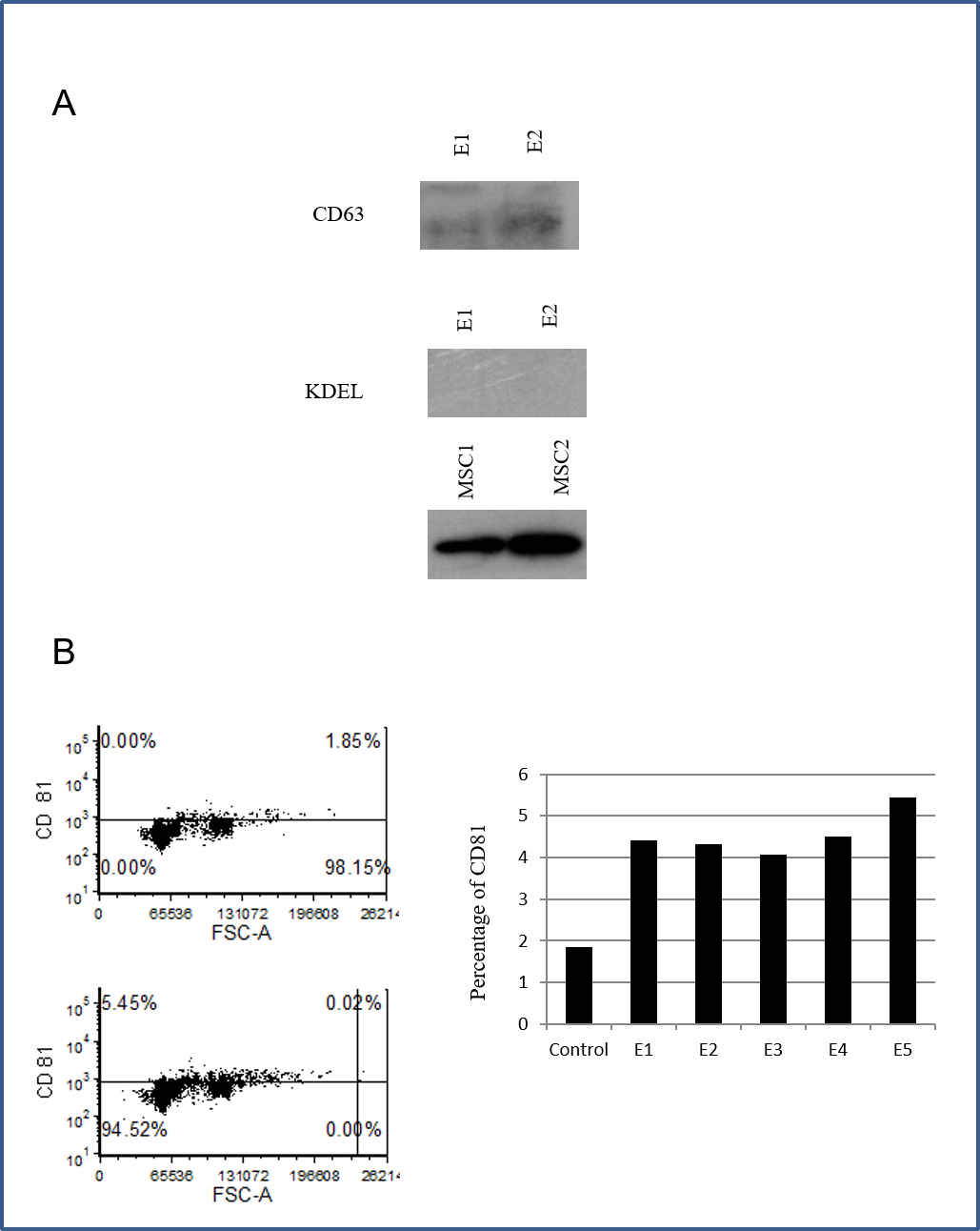

Supplement: Supplementary file 7 [file Image_3.tif]

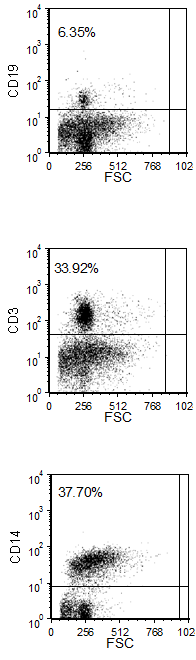

Supplement: Supplementary file 8 [file Image_4.TIF]

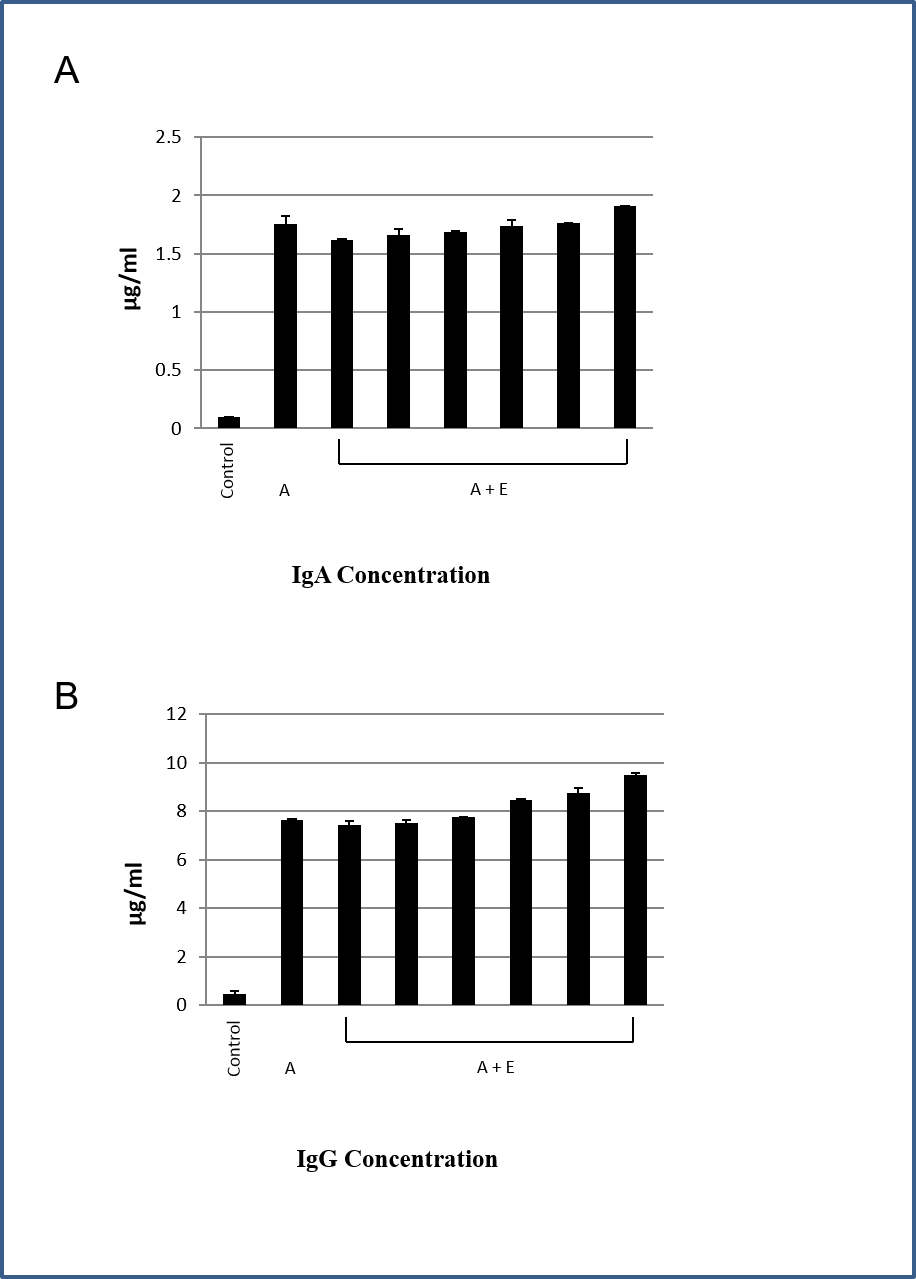

Supplement: Supplementary file 9 [file Image_5.TIF]
